# Psychosis Early Intervention Across the Life Span: A Service Perspective

**DOI:** 10.1007/s10597-022-01004-x

**Published:** 2022-08-03

**Authors:** Kathryn M. Taylor, Ela Orucu, Sunil Nandha, Matteo Cella

**Affiliations:** 1 Department of Psychology, Institute of Psychiatry, Psychology, and Neuroscience, King’s College London, De Crespigny Park, London, SE5 8AF UK; 2 South London and Maudsley NHS Foundation Trust, London, UK

**Keywords:** Early intervention, Mental health services, Psychological therapies, Psychosis, Schizophrenia

## Abstract

Previously youth-orientated, UK early intervention in psychosis (EI) services expanded care in 2016 to adults of any age. To compare EI care offering, clinical characteristics, and potential benefits for under-35s and over-35s, an observational study considering anonymised data for users of London-based EI services between April 2016 and December 2019 was conducted. Descriptive statistics and between groups comparisons are reported. The analysis considered 692 service users (32.5% over-35). Over-35s were more likely to be female, of poorer physical health, with severer problems at intake (Health of the Nation Outcome Scale, HoNOS). Under-35s had poorer appointment attendance, required increased use of inpatient facilities, and demonstrated greater risks to themselves and others. At discharge, HoNOS ratings indicated improvements for both groups. Over-35s constitute a considerable proportion of EI service-users, their care may involve less crisis management, more recovery-oriented intervention and physical health needs consideration. Care offering should reflect these needs.

## Introduction

Psychosis incidence is most common in late adolescence to early adulthood, though continues to emerge throughout the lifespan, with up to 30% of cases having an onset over the age of 35 years (Häfner et al., 1993, 1998). Early intervention in psychosis (EI) services originated as youth-orientated (Fusar-Poli et al., 2017; McGorry et al., 2007). In England, EI services were created to offer care to service users under the age of 35, but in April 2016 the maximum age restriction was expanded to 65 years (National Institute for Health and Care Excellence, 2016). Contributing towards the rationale for age inclusivity are findings indicating that individuals with later onset psychosis also experience an extended, potentially longer, duration of untreated psychosis (DUP; Clay et al., 2018; Lappin et al., 2016; Selvendra et al., 2014) and equally poor clinical or functional outcomes (Lappin et al., 2016).

In the UK it is estimated that since the change in age restrictions, between 22 and 30% of referrals to EI services are aged over 35 years (Adamson et al., 2018; Clay et al., 2018; Greenfield et al., 2018; Häfner et al., 1998). Concerns were raised that the shift away from the EI youth-focus may adversely affect the quality of the care provided for younger service users, and that the higher caseload may create a strain on the service (Varregoso et al., 2018). There is currently limited evidence of the acceptability and efficacy of the youth-focused provision of care from EI services for over 35s.

Evidence suggests that individuals with later psychosis onset may have different clinical complexities including comorbid physical health difficulties, higher levels of unemployment, more dependents, and a higher likelihood of affective presentations (Chen et al., 2018; Greenfield et al., 2018; O’Driscoll et al., 2019; Selvendra et al., 2014). Removing age-related criteria for EI services may consequently require substantial adaptation of the care provided and of staff skillset. Evaluation studies have begun to explore age-related differences in EI service care provision. One study found that over 35s required more contact from healthcare professionals (e.g. care coordinators), interpreted to indicate more complexity, and greater clinical and social needs (Jagger et al., 2019). Another found no differences in clinical outcomes, despite variation in social factors between the age groups (O’Driscoll et al., 2019).

Currently there is limited evidence on how service users’ age may impact clinical presentations and needs. Given the limited resources available to EI services, it is important to ensure the treatment available is appropriate, beneficial, and acceptable to those aged over 35. This study aims to provide evidence to address this, to support decisions around resource use and the continued development of EI services. The objective of this study was therefore to compare the care offering, clinical characteristics, and potential benefits of EI services for individuals aged under-35 to those aged over-35.

## Methods

For this observational study, we considered all clinical data available from four EI services, part of the South London and Maudsley (SLaM) NHS Trust. The Trust’s Clinical Record Interactive Search (CRIS) interface (Stewart et al., 2009) was used to extract de-identified information from the electronic clinical record system. CRIS is a well-established system and has been used for multiple research studies (Colling et al., 2020; Oduola et al., 2019; Roberts et al., 2016). Data was extracted on 2nd December 2019. The research protocol was reviewed and approved by the SLaM Trust Psychosis Clinical & Academic Group and CRIS oversight committee and given favourable opinion. All data considered were anonymised.

### Participants

Participants were service users under the care of the EI services, with a care spell between April 2016 and December 2019. All service users had experienced at least one episode of psychosis. As EI services provide targeted interventions following a first episode of psychosis for approximately two years, extraction dates allowed for service users to have had a care spell period of at least two years (unless discharged prior). If service users had not yet been discharged from the EI team, data was extracted from the accepted date up until the date of data extraction (2nd December 2019). For service users with multiple, short care spells, we considered only those with a first episode of care lasting one year or more.

### Measures

The following were extracted for each service user: (i) demographic information as recorded at intake, including age, gender, and ethnicity; (ii) the Index of Multiple Deprivation (IMD; Smith et al., 2015), based on the service user’s home address, as an indication of relative deprivation; (iii) primary and secondary diagnoses at discharge; (iv) the Health of the Nation Outcome Scale (HoNOS; Wing et al., 1998), as a measure of social functioning levels (assessed at intake and discharge); (v) historic and current risk was evaluated with an assessment tool, completed at intake and approximately every six months while under EI care; (vi) treatment characteristics during the care spell, regarding service use, medication and appointment attendance with different professional groups; (vii) information regarding offers of and attendance at UK clinical guideline recommended therapies (Cognitive Behavioural Therapy (CBTp) and Family Intervention (FIp); National Institute for Health and Care Excellence, 2014).

### Analysis

Descriptive statistics are reported. For continuous variables, between groups differences were assessed using independent samples *t*-tests, or Mann Whitney U tests where normality assumptions were violated. Paired samples *t*-tests, or Wilcoxon Signed-Rank tests if indicated, were used to assess change over time. Pearson’s chi-squared tests were used to assess differences between categorical data. Throughout these analyses, age-group (i.e., under or over 35) was the independent variable. Cases with relevant missing variables were excluded from each analysis. Analyses were conducted using SPSS 26.

## Results

The search identified 813 service users accepted by the EI teams within the time period considered. Of these, 121 were excluded due to having: multiple EI episodes where the extracted episode lasted less than a year (*n* = 49) or the first EI episode started prior to April 2016 (*n* = 45); not having been offered treatment or care by the EI team (i.e., no face-to-face event either attended or not attended; *n* = 16) or being accepted and discharged on the same day (*n* = 11). A total of 692 service users were considered in the analysis.

### Socio-demographic Characteristics

Details of socio-demographic characteristics are presented in Table 1. For analyses, participants were divided into two age groups: 467 (67.5%) aged 17–34 years and 225 (32.5%) aged 35–64 years. While the two age groups did not differ significantly in ethnicities or relative deprivation, a significantly larger proportion of over-35-year-olds (O-35s) were female (*X*^*2*^(1, *N* = 692) = 29.97, *p* < 0.001).

**Table 1:** Descriptive statistics and results of chi-square test for sociodemographic and clinical characteristics by age group

Sociodemographic characteristic	Under 35 years (*n* = 467) *n* (%)	Over 35 years (*n* = 225) *n* (%)	*X*^2^ value
Age in years [Mean (SD)]	23.94 (4.77)	45.50 (7.90)	–
Gender			
Male	319 (68.31%)	105 (46.67%)	29.97***
Female	148 (31.69%)	120 (53.33%)	
Ethnicity			
Black/African/Caribbean/Black British	208 (44.54%)	78 (34.67%)	9.22
White	147 (31.48%)	77 (34.22%)	
Any other ethnic group	35 (7.49%)	25 (11.11%)	
Not known/Not stated	27 (5.78%)	28 (12.44%)	
Asian/Asian British	35 (7.49%)	15 (6.67%)	
Mixed/Multiple Ethnic Groups	15 (3.21%)	2 (0.89%)	
IMD decile			
1st (Most deprived 10%)	31 (6.64%)	16 (7.11%)	11.02
2nd	146 (31.26%)	69 (30.67%)	
3rd	97 (20.77%)	56 (24.89%)	
4th	75 (16.06%)	32 (14.22%)	
5th	48 (10.28%)	25 (11.11%)	
6th	24 (5.14%)	13 (5.78%)	
7th	6 (1.28%)	2 (0.89%)	
8th	10 (2.14%)	0 (0.00%)	
9th	4 (0.86%)	6 (2.67%)	
10th (Least deprived 10%)	2 (0.43%)	0 (0.00%)	
Not known	24 (5.14%)	6 (2.67%)	
Early intervention service^a^			
Croydon—COAST	121 (67.22%)	59 (32.78%)	3.22
Lewisham—LEIS	117 (69.64%)	51 (30.36%)	
Lambeth—LEO	116 (62.70%)	69 (37.30%)	
Southwark—STEP	113 (71.07%)	46 (28.93%)	
Medication type			
Antipsychotics	447 (95.72%)	199 (88.44%)	12.94***
Antipsychotics: Clozapine	36 (7.71%)	7 (3.11%)	5.51**
Anxiolytics	266 (56.96%)	93 (41.33%)	14.85***
Antidepressants	177 (37.90%)	101 (44.89%)	3.09
Mood Stabilisers	60 (12.85%)	9 (4.00%)	13.24***

*IMD* Index of Multiple Deprivation, *COAST* Croydon Outreach and Assertive Support Team, *LEI* Lewisham Early Intervention Team, *LEO* Lambeth Early Onset, *STEP* Southwark Team for Early Psychosis

*p < 0.05; **p < 0.01; ***p < 0.001

^a^Reported as percentage within each EI service

### Clinical Characteristics

#### Diagnoses

Across the entire sample the most common primary diagnoses at discharge were Schizophrenia, Schizotypal and Delusional Disorders (ICD-10 F20-29; 67.7%), or Mood Disorders (F30-39; 10.0%). The remainder had another diagnosis (15.6%), or none recorded (6.6%). When compared by ICD-10 diagnostic clusters (e.g. F10-19; F20-29), there was no significant difference in diagnosis prevalence between the age groups (*X*^2^ (9, *N* = 692) = 7.24, *p* = 0.612), even when considering only those who had a completed EI care spell (*X*^2^ (9, *N* = 449) = 5.15, *p* = 0.821). Thirty-five (5.1%) had at least one secondary diagnosis recorded; this did not differ between age groups.

#### Medication

As shown in Table 1, a greater proportion of under-35-year-olds (U-35s) compared to O-35s were prescribed antipsychotics (*p* < 0.001), anxiolytics (*p* < 0.001) and mood stabilisers (*p* < 0.001), but not antidepressants (*p* = 0.079).

#### Clinical Risk

Risk events (as recorded in clinical notes) were compared across the whole sample, 405 of whom (58.5%) had at least one significant risk event reported during the episode. A non-significant larger proportion of U-35s had at least one risk event reported (61%, *n* = 285) than O-35s (53%, *n* = 120; *X*^*2*^ (1, *N* = 692) = 3.70, *p* = 0.054). Events relating to violence and aggression (Mann–Whitney *U* = 42671.00, *p* < 0.001), self-harm and suicide *(U* = 46344.50, *p* < 0.001), other risk *(U* = 45834.50, *p* = 0.001) and physical health *(U* = 56503.50, *p* = 0.021) were more frequently reported for U-35s. Only 42.8% (U-35 42.2%; O-35 44.0%) had a complete Risk Assessment Tool at intake, with 19.2% (U-35 18.8%; O-35 20.0%) having none completed during the care spell. Of those with an intake baseline Risk Assessment Tool recorded, risk related to violence and aggression was more likely to be reported for U-35s than O-35s (57.9% vs. 32.3%; *X*^2^ (1, *N* = 296) = 17.20, *p* < 0.001), whereas risk related to physical health was more common for O-35s (14.7% vs. 33.3%; *X*^2^ (1, *N* = 296) = 13.79, *p* < 0.001). There were no group differences in the other risk domains including suicide and self-harm, safeguarding adults and children, neglect, and radicalisation.

### Treatment Characteristics

#### Service Use

EI episode duration ranged from 8 to 1,337 days (median = 777, IQR = 619) and did not differ significantly by age group (*p* > 0.2; see Table 2 for details of duration of service use by age group). U-35s were more likely to be an inpatient when accepted onto the EI caseload (39.8% vs. 25.7%; *X*^*2*^(1, *N* = 692) = 13.13, *p* < 0.001), require an inpatient admission during the episode (57.8% vs. 38.6%; *X*^*2*^(1, *N* = 692) = 22.29, *p* < 0.001) and be detained under the UK Mental Health Act (47.3% vs. 28.0%; *X*^*2*^(1, *N* = 692) = 23.43, *p* < 0.001). Inpatient admissions were more frequent for U-35s (Mdn = 1.00 admission (IQR = 1.00) vs. 0.00 (1.00); *U* = 40108.50, *p* < 0.001), and lasted longer (see Table 2; *p* = 0.001). Around half (52.2%) of the whole sample required at least one day of Home Treatment Team (HTT) input during the EI episode, ranging from 1 to 128 days (Mdn = 19, IQR = 26). While total duration of HTT involvement did not differ significantly by age group (*p* > 0.9), U-35s were more likely to require the service (55.5% vs. 45.3%; *X*^*2*^ (1, *N* = 692) = 6.24, *p* = 0.012).

**Table 2:** Descriptive statistics and Mann–Whitney *U* test results for service use duration in days by age group

Care type	Under 35				Over 35				*U*	*Z*
	*n*	Mean (SD)	Median (IQR)	Skew (SE)	*n*	Mean (SD)	Median (IQR)	Skew (SE)		
EI episode	467	689.01 (369.61)	780.00 (624.00)	− 0.23 (0.11)	225	661.12 (359.83)	767.00 (580.50)	− 0.29 (0.16)	49812.50	− 1.11
Inpatient admission	270	93.76 (125.85)	50.00 (90.25)	3.00 (0.15)	87	48.21 (53.72)	27.00 (55.00)	2.24 (0.26)	9004.50	− 3.27**
MHA section	221	93.15 (119.88)	55.00 (92.00)	2.95 (0.16)	63	52.17 (53.48)	37.00 (60.00)	2.00 (0.30)	5593.50	− 2.38*
Home Treatment Team	259	26.56 (24.00)	19.00 (26.00)	1.78 (0.15)	102	25.87 (22.03)	19.50 (28.25)	1.49 (0.24)	13196.50	− 0.01

*SD* standard deviation, * IQR* interquartile range, *SE* standard error, *U* Mann–Whitney test coefficient, *Z* standardised test statistic, *EI* early intervention, *MHA* mental health act

*p < 0.05; **p < 0.01; ***p < 0.001

Most service users attended at least one appointment for care coordination (91.5%), medical care (e.g., consultation with a psychiatrist; 87.1%), psychological therapy (52.7%), and nursing (49.7%). A smaller proportion attended at least one appointment with a support worker (10.0%), occupational therapist (9.2%), social worker (7.5%) or vocational worker (6.8%). Average appointment attendance rates were lower for U-35s for care coordination (U-35: Mdn = 85.7% (IQR = 25.6%); O-35: 90.4% (22.9%); Mann–Whitney *U* = 51362.50, *p* = 0.011), psychological therapy (76.7% (39.0%) vs. 87.5% (32.1%); *U* = 18029.50; *p* = 0.001) and vocational support (61.1% (25.0%) vs. 100.0% (36.4%); *U* = 314.00, *p* = 0.019). Attendance did not differ significantly between age groups for any other appointment type.

#### Engagement in Psychological Therapy

Two-hundred and thirty-three individuals (33.7%) attended at least one CBTp session (mean 11; median 8; SD 9.4; IQR 14). One hundred and thirty-five (19.5%) attended at least one FIp session (mean 5.5; median 3; SD 5.3; IQR 6). A small, equivalent proportion of both age groups attended the UK minimum recommended dose of ≥ 10 FIp sessions (U-35: 3.6%; O-35: 3.6%; (*X*^2^ (1, *N* = 692) = 0.003, *p* = 0.955) and ≥ 16 CBTp sessions (U-35: 9.0%; O-35: 11.6%; *X*^2^ (1, *N* = 692) = 1.125, *p* = 0.289). Frequency of non-attended and cancelled sessions labelled as CBTp or FIp did not differ between the age groups (*p* > 0.7). Therapeutic modality was not specified for 1,326 psychological therapy events, recorded for 43.2% (*n* = 299) of the service users considered. It was not possible to determine if these events were CBTp or FIp sessions.

#### Clinical Outcomes

Functional difficulties captured by the HoNOS are presented in Table 3. HoNOS scores were recorded for 47.3% of service users at intake and 40.2% at discharge, 22.1% had ratings recorded for both time points. Across the whole sample, there was a significant reduction in functioning difficulties from baseline (Mdn = 10.0, IQR = 9.0) to discharge (Mdn = 6.00, IQR = 8.25; *Z* = − 6.07, *p* < 0.001). Both groups showed a significant reduction in levels of difficulty over the EI care period.

**Table 3:** Descriptive statistics, Wilcoxon signed-rank test and Mann–Whitney* U* test results for HoNOS scores at baseline and discharge by age group

HoNOS scale item	Under 35					Over 35					Between groups comparison			
	Baseline (*n* = 222)		Discharge (*n* = 204)		Wilcoxon signed-rank *Z* (*n* = 112)	Baseline (*n* = 105)		Discharge (*n* = 74)		Wilcoxon signed-rank *Z* (*n* = 41)	Baseline		Discharge	
	Mean (*SD*)	Median (IQR)	Mean (SD)	Median (IQR)		Mean (SD)	Median (IQR)	Mean (SD)	Median (IQR)		*U*	*Z*	*U*	*Z*
Agitated behaviour	0.77 (1.01)	0.00 (1.00)	0.35 (0.68)	0.00 (0.75)	− 3.61***	0.58 (0.82)	0.00 (1.00)	0.32 (0.72)	0.00 (0.00)	− 1.71	10708.00	− 1.33	7296.00	− 0.57
Self-injury	0.18 (0.57)	0.00 (0.00)	0.10 (0.42)	0.00 (0.00)	− 2.42*	0.24 (0.66)	0.00 (0.00)	0.05 (0.28)	0.00 (0.00)	− 2.05*	11344.00	− 0.68	7371.00	− 0.74
Drinking/drugs	0.68 (1.04)	0.00 (1.00)	0.57 (0.97)	0.00 (1.00)	− 1.21	0.53 (0.98)	0.00 (1.00)	0.42 (0.89)	0.00 (0.00)	− 0.11	10647.00	− 1.36	6881.50	− 1.42
Cognitive problems	0.62 (0.99)	0.00 (1.00)	0.38 (0.72)	0.00 (1.00)	− 2.53*	0.69 (0.94)	0.00 (1.00)	0.39 (0.74)	0.00 (1.00)	− 2.75**	11024.00	− 0.84	7530.50	− 0.04
Physical illness	0.34 (0.73)	0.00 (0.00)	0.29 (0.70)	0.00 (0.00)	− 0.96	0.85 (1.13)	0.00 (2.00)	0.81 (1.05)	0.00 (1.50)	− 0.59	8937.50	− 4.23***	5296.50	− 4.78***
Hallucinations	1.68 (1.36)	2.00 (3.00)	0.73 (1.04)	0.00 (1.00)	− 5.31***	2.04 (1.24)	2.00 (2.00)	0.95 (1.05)	1.00 (2.00)	− 3.48**	9884.50	− 2.22*	6609.00	− 1.76
Depressed mood	1.09 (1.01)	1.00 (2.00)	0.71 (0.87)	0.00 (1.00)	− 3.88***	1.28 (1.01)	1.00 (1.00)	0.82 (0.88)	1.00 (1.25)	− 1.94	10448.00	− 1.58	6958.00	− 1.08
Other mental health difficulties	1.55 (1.17)	2.00 (2.00)	0.97 (1.06)	1.00 (2.00)	− 3.82***	1.59 (1.16)	2.00 (1.00)	1.04 (1.05)	1.00 (2.00)	− 3.14**	11464.00	− 0.11	7242.00	− 0.55
Relationship problems	1.19 (1.13)	1.00 (2.00)	0.82 (0.99)	0.50 (1.00)	− 2.73**	1.45 (1.16)	1.00 (1.50)	0.81 (0.99)	0.50 (1.00)	− 1.96*	10169.00	− 1.93	7508.50	− 0.07
Daily living problems	0.84 (1.05)	0.00 (2.00)	0.54 (0.91)	0.00 (1.00)	− 2.74**	0.89 (1.05)	1.00 (2.00)	0.74 (0.98)	0.00 (1.25)	− 0.76	11264.00	− 0.53	6679.00	− 1.73
Living conditions problems	0.59 (0.96)	0.00 (1.00)	0.55 (0.93)	0.00 (1.00)	− 0.91	0.78 (1.11)	0.00 (1.00)	0.62 (0.87)	0.00 (1.00)	− 0.25	10402.50	− 1.68	7021.00	− 1.05
Occupational problems	1.12 (1.08)	1.00 (2.00)	0.85 (1.01)	0.00 (2.00)	− 2.19*	1.38 (1.17)	1.00 (2.00)	0.77 (0.88)	0.00 (2.00)	− 2.79**	10221.50	− 1.87	7370.00	− 0.33
Total	10.67 (6.26)	10.00 (9.00)	6.87 (6.03)	5.00 (8.00)	− 5.00***	12.29 (6.65)	11.00 (9.00)	7.75 (5.82)	6.00 (8.25)	− 3.48***	9892.00	− 2.21*	6725.50	− 1.39

Scale item scores can range from 0 to 4. Total scores can range from 0 to 48. Baseline ratings include first rating within six months of acceptance into EI service. Discharge ratings include last rating within six months prior to discharge from EI service

*SD* standard deviation, *IQR* interquartile range, *U* Mann–Whitney test statistic, *Z* standardised test statistic

*p < 0.05; **p < 0.01; ***p < 0.001

## Discussion

This study sought to explore differences in clinical presentation and use of EI services amongst service users with early versus late psychosis onset, using routine data from four EI services in south London, UK.

We found gender distribution to be uneven in the two age groups considered; further suggesting that a later psychosis onset may be more common in women (Häfner et al., 1998; Jagger et al., 2019; O’Driscoll et al., 2019; Selvendra et al., 2014). Previous age restrictions for EI services were therefore likely gender inequitable and disproportionately disadvantaged women. In contrast to previous studies, which found O-35s to be more likely to present with affective psychosis, we did not find differences in diagnosis between the age groups (Clay et al., 2018; Jagger et al., 2019; O’Driscoll et al., 2019). Additionally, we did not find differences in comorbid mental health secondary diagnoses. HoNOS score ratings did, however, indicate more severe difficulties at baseline for service users O-35. This may be explained by the divergent routes to accessing EI: younger service users were more likely to have commenced treatment through an inpatient admission prior to entry to EI.

HoNOS ratings related to physical health were poorer for O-35s, as might be expected in an older population. Poor physical health is common for people living with psychosis (Stubbs et al., 2016). Studies suggest that people with this diagnosis have a 10–25 year reduction in life expectancy (Laursen et al., 2012). Regular physical health checks, monitoring and appropriate treatment are described as one of the key components of the EI service (National Institute for Health and Care Excellence, 2016), and must be a priority, particularly for those O-35. While only a small proportion of both age groups had HoNOS data available at pre- and post- service use, those who did showed significant improvement in HoNOS scores overall, and for the individual items including self-injury, cognitive problems, hallucinations, other mental health problems, relationship problems and occupational problems. This finding is in line with previous research suggesting EI services are beneficial (Bird et al., 2010; Correll et al., 2018; Fusar-Poli et al., 2017), and studies demonstrating improved outcomes following EI service use for both under and over 35s (Lappin et al., 2016; Lasalvia et al., 2017; O’Driscoll et al., 2019).

U-35s required more crisis management, with more incidents reported in terms of risk to self and aggression towards others, longer and more frequent inpatient admissions and a greater requirement for HTT input. This is consistent with prior service evaluations (Lappin et al., 2016; O’Driscoll et al., 2019), and typical of observed age-related differences in risk in the wider population (e.g., Bachmann 2018; McManus et al., 2019). The higher occurrence of risk behaviours and need for crisis support in U-35s may likely be explained by the developmental differences of the age groups, for example in impulsivity, emotion regulation, and psychosocial maturity. Additionally, O-35s were more engaged with the EI service showing better attendance for care coordination, psychological therapy, and vocational support appointments. While previous research has interpreted more frequent care coordination contact by O-35s as indicative of higher clinical complexity (Jagger et al., 2019), this could also reflect differences in coping styles and help-seeking behaviour; with individuals O-35 seeking support from EI services more effectively before reaching crisis. This has implications for interventions, such as psychological therapies, which may require a greater focus on promoting self-management and prompt help-seeking for U-35s. To inform crisis planning and intervention focus, future research should examine differences in coping and help-seeking behaviour between early and late-onset groups.

A small proportion of both age groups received the NICE recommended number of 10 or more FIp sessions (3.6%) and 16 or more CBTp sessions (9.8%). This is in line with, or slightly higher than, previous implementation estimates (Haddock et al., 2014; Ince et al., 2016). Barriers to access still exist and appear to impact both age groups equally. Possible barriers may include long waiting lists, work pressure and high caseloads, lack of specialist workers, and staff knowledge and attitudes (Prytys et al., 2011). Additionally, some individuals may attend fewer sessions for positive reasons, such as achieving therapy goals more quickly, or due to other targeted, low-intensity psychological interventions effectively improving outcomes, in line with the interventionist-causal model approach (Freeman, 2011). This study also attempted to identify ‘offers’ made for NICE recommended therapies, by exploring a subset of notes from individuals’ electronic health records to identify if these therapies were offered and declined, not offered, or offered and on a waiting list to be delivered. Due to record inconsistency, it was not possible to draw any firm conclusion. Reporting standards should be introduced to facilitate the evaluation of adherence to guidelines for timely access to interventions such as the UK Access and Waiting Time Standard (National Institute for Health and Care Excellence, 2016).

The results of this evaluation rely on the accuracy of record-keeping, which may be subject to human error. While the data was cleaned, checked for systematic errors, and errors resolved, human errors in record keeping are still possible. However, these should be distributed at random and unlikely to bias this study’s conclusions. It is notable that routine outcome measures, such as the HoNOS, as well as clinical tools, such as risk assessments, were not available for a significant proportion of the sample. This limits the scope and generalisability of this evaluation’s findings. It would be important for services to ensure higher completion rate of such measures, to not only ensure the provision of safe and effective care, but also to facilitate future analyses and monitoring of outcomes.

Attendance rates reflected contact with professionals within the EI team only, therefore omitting support received from other community or inpatient services. Due to longer durations of inpatient admission, this may have somewhat disproportionately represented contact for U-35s. Nonetheless, the reported findings present an accurate representation of EI service usage. Future work should examine differences in duration, content, and quality of clinical contacts to better determine adherence to clinical guidelines. This may further identify differences in the treatment provision and compliance in the two age groups and may help to establish differential care pathways.

### Conclusions

Findings from this study indicate that the considered UK EI services are effectively adapting to accommodate the needs of U-35s and O-35s. As such, the need for distinct services or provision of care for these age groups is not indicated. It is notable, however, that only a minority of individuals in both age groups attending these specialist services received a dose of psychological therapies in line with UK guidelines, and increasing access for all service users must remain a priority. This study suggests that differences may exist in care uptake and presentation to services between those experiencing psychosis at different life stages, and these must be considered in care planning and delivery.

## Data Availability

Research data are not shared.
